# Swooping in the Suburbs; Parental Defence of an Abundant Aggressive Urban Bird against Humans

**DOI:** 10.3390/ani3030754

**Published:** 2013-08-13

**Authors:** Daniel Lees, Craig D. H. Sherman, Grainne S. Maguire, Peter Dann, Adam P. A. Cardilini, Michael A. Weston

**Affiliations:** 1 Centre for Integrative Ecology, Faculty of Science, Engineering and the Built Environment, Deakin University, 75 Pigdons Rd., Waurn Ponds, VIC 3216, Australia; E-Mails: craig.sherman@deakin.edu.au (C.D.H.S.); a.cardilini@gmail.com (A.P.A.C.); 2 BirdLife Australia, Suite 2-05, The Green Building, 60 Leicester Street, Carlton, VIC 3052, Australia; E-Mail: grainne.maguire@birdlife.org.au; 3 Research Department, Phillip Island Nature Parks, P.O. Box 97, Cowes, Phillip Island, VIC 3922, Australia; E-Mail: pdann@penguins.org.au; 4 Centre for Integrative Ecology, Faculty of Science, Engineering and the Built Environment, Deakin University, 221 Burwood Highway, Burwood, VIC 3125, Australia; E-Mail: mweston@deakin.edu.au

**Keywords:** parental defence, human disturbance, swooping, human-wildlife conflict, hatching success

## Abstract

**Simple Summary:**

We studied the defensive behaviour of 94 pairs of nesting Masked Lapwings, *Vanellus miles*, in response to two types of human stimuli: a pedestrian and a person pushing a lawn mower. We also examined the effectiveness of a commonly promoted deterrent to swooping (the presence of mock eyes placed on the back of a person’s head) for each stimulus type. Masked Lapwings responded more aggressively to a person pushing a lawn mower compared with pedestrians. Birds also remained closer to the nest in the presence of a lawn mower. The presence of eye stickers on the back of a pedestrian’s head decreased swooping behaviour; however, the presence of eye stickers worn by a person pushing a lawn mower increased swooping behaviour.

**Abstract:**

Masked Lapwings, *Vanellus miles*, often come into ‘conflict’ with humans, because they often breed in close proximity to humans and actively defend their ground nests through aggressive behaviour, which typically involves swooping. This study examined whether defensive responses differed when nesting birds were confronted with different human stimuli (‘pedestrian alone’ *vs*. ‘person pushing a lawn mower’ approaches to nests) and tested the effectiveness of a commonly used deterrent (mock eyes positioned on the top or back of a person’s head) on the defensive response. Masked Lapwings did not swoop closer to a person with a lawn mower compared with a pedestrian, but flushed closer and remained closer to the nest in the presence of a lawn mower. The presence of eye stickers decreased (pedestrians) and increased (lawn mowers) swooping behaviour. Masked Lapwings can discriminate between different human activities and adjust their defensive behaviour accordingly. We also conclude that the use of eye stickers is an effective method to mitigate the human-lapwing ‘conflict’ in some, but not all, circumstances.

## 1. Introduction

Depredation is a major influence on the reproductive success of many bird species [1,2]. A depredation event often results in the loss of entire clutches or broods and can cause breeding pairs to fail to successfully fledge offspring during a breeding season [2]. Thus, the defence of reproductive investment has evolved as an important adaptation for maximising an individual’s reproductive success. However, there is a trade-off between the risks associated with parental defence and future reproduction [3]. In order to maximise the number of offspring an individual produces over its lifetime, it needs to balance the risk to itself associated with defence against the enhanced survival of current offspring. Based on the risk to the parent, and the investment in current offspring and offspring survival, it is possible to predict an optimum level of parental defence [4,5,6]. Avian parental defence is diverse and can involve alarm calling, injury feigning, ‘false’ incubating or brooding, chasing, attacking or harassing a predator alone, with conspecifics or other species [5]. Arguably, the most risk-prone form of parental defence is chasing or attacking a predator, but it can also be the most effective form of defence [5,7]. 

Each year, throughout the world, people are injured by bird attacks from various species, some of which result in serious, often permanent, injury [8,9,10,11,12]. Bird attacks on people represent one of the most prominent human-wildlife conflicts [13]. In Australia, one common swooping bird is the Masked Lapwing (*Vanellus miles*) [14]. The Masked Lapwing (henceforth ‘lapwing’) is renowned for its defensive behaviour and often aggressively swoops potential threats to its eggs or young [14,15,16]. Lapwings also use other forms of parental defence, such as injury feigning, open wing aggression displays and alarm calling [16]. Lapwings are often persecuted by humans and widely regarded as ‘problem wildlife’. For example, there have been reports of ground maintenance staff deliberately attacking adult lapwings with brush-cutters (presumably in response to the swooping behaviour), destroying nests and eggs (e.g., by mowing them) and maiming parents [17]. Given that at least some birds can discriminate between people engaged in different activities and that birds often respond to humans using anti-predator responses [18], it may be that lapwings are especially aggressive to humans associated with activities or equipment recognised by birds as representing greater threats to eggs and young. Indeed, recent research has shown that lapwings nesting in urban environments are more aggressive to people than those nesting in rural areas [19].

Management authorities offer advice as to how to manage swooping by problem birds, including: avoiding the area, wearing a bicycle helmet, carrying a stick with a flag on the end above their heads and, very commonly, the wearing of realistic looking eye stickers on the back of hats or helmets [14,20]. The use of eye stickers exploits the ability of many species to monitor the gaze of their adversaries, presumably to monitor the risk of attack or counter attack [14,21,22]. Jones and Thomas [8] suggest, but do not test, the success of eye stickers in minimising attacks from nesting Australian Magpies (*Gymnorhina tibicen*) and suggest that eye stickers are effective at deterring swooping aggression on pedestrians, but not cyclists. While only 18% of nesting Australian Magpie pairs were aggressive towards humans, 73% of lapwing pairs swooped humans [15,23]. Although lapwings are less common than Australian Magpies in many urban areas, they are very common and are widespread in some suburbs [16,19] and frequently interact with humans, with the potential to cause injury. Thus, understanding lapwing swooping and ways to mitigate it is a management imperative.

This study aims to determine if lapwings display higher levels of aggressive behaviour (nest defence behaviour) towards ground maintenance staff engaged in lawn mowing compared with the general public and whether eye stickers are an effective deterrent to aggressive swooping. Specifically, this study aims to examine:

1. if lapwings can discriminate between different human activities, specifically between pedestrians and ground maintenance personnel mowing lawns;
2. if the presence of eye stickers (mounted on the back of a person’s head) represents an effective way of managing lapwing attacks on people; and,
3. whether hatching success is influenced by parental defence. More aggressive parents may experience improved reproductive success [24,25], and this may reinforce, or even select for, aggressiveness.

Specifically, we predicted lapwings would display higher levels of aggression towards a person pushing a lawn mower than a pedestrian, because the former represents a greater risk to eggs or young. We also predicted that eye stickers would reduce, and perhaps effectively mitigate, lapwing swooping. Finally, we predict that more aggressive lapwing pairs will experience greater reproductive success in the form of hatching success.

## 2. Methods

The lapwing is particularly common in urban areas on Phillip Island, Victoria, Australia (38°29.112'S, 145''13.787'E) [16,19,26]. It can be classified as an urban exploiter [19], which is unusual for a ground-nesting species with precocial and nidifugous chicks, which remain with the adults on defended territories until fledging [16,27,28]. The chicks commence feeding within hours of hatching and rely on their parents for protection (alarm signals and aggression) and thermoregulation (brooding) until fledging at approximately six to seven weeks of age [16]. 

### 2.1. Study Area

Fieldwork was conducted on Phillip Island between May 22 and September 15, 2012 (in southern Australia, lapwings are winter breeders [28]). Phillip Island was chosen, because it has an abundant population of lapwings, some of which have previously been individually marked as part of a monitoring and research program [15,19] (pilot analyses revealed that previous banding did not influence bird responses). The island was systematically searched for nests throughout the breeding season. Particular attention was paid to known breeding locations around the townships of Cowes, Rhyll and Surf Beach. Nests were located by searching by vehicle or by foot [19].

### 2.2. Evoking Defensive Behaviour

Standardised experimenter ‘approaches’ to nests were used to evoke defensive behaviour. In order to assess the level and type of parental defence of nesting adults in regards to different perceived threats from humans, nests were randomly assigned to one of four approach types (henceforth ‘stimuli’). These were: (1) pedestrian, (2) pedestrian with eye stickers, (3) ground maintenance worker with lawn mower and (4) ground maintenance worker with lawn mower and eye stickers. Each nest only received one type of approach, and there were no multiple approaches to the same nest.

Each approach type was standardised and made by D. Lees. The pedestrian approach involved wearing a black vest, dark pants, rubber boots and a grey cap. The pedestrian with eye stickers was clothed in the same way, but had realistic looking eye stickers (offered by management agencies as deterrents for swooping and mounted, facing backwards on the back of the grey cap [14]). The ground maintenance worker treatment involved the wearing of a fluorescent yellow vest, dark pants, rubber boots, grey cap and a lawn mower. The lawn mower’s engine had been removed and replaced by two 2-watt speakers playing a recording of an operating lawn mower at a realistic volume. The ground maintenance worker with eye stickers was identical, but eye stickers were mounted on the back of the cap. The ground maintenance worker treatments (including the fluorescent yellow vest) were designed to mimic actual ground maintenance staff on Phillip Island who had been observed during a pilot study. This study does not seek to elucidate the specific aspects of each stimuli which may alter lapwing responses. 

### 2.3. Indexing Parental Defence

Parental defence was measured by delivering standardised experimental approaches (in the absence of predators). The investigator slowly, but deliberately, walked towards the nest. Once the investigator arrived at the nest, the investigator stood motionless, facing the same direction as the approach, for two minutes (standard two minute observation period, henceforth ‘SOP’) and continued to record behavioural displays (for a description of displays, see [16]). Starting (distance between the investigator’s initial observation position and the focal incubating bird [29]) and response distances (first change in incubator behaviour [18]) were recorded using a laser rangefinder. Separation distance was always measured from the person to the bird; during lawn mower approaches, lapwing responses were always focused on the person, not the lawn mower itself. A car window or tripod mounted video camera (GoPro HD Hero 2) recorded lapwing behaviour for the duration of the approach. Before an approach began, a brief observation period included: the recording of the location and behaviour of both birds (although occasionally only the incubating bird was present), as well as the recording of habitat type (‘suburban’, any area with built up human residences within 50 metres of the nest, bound by fences; or ‘rural’, any area outside suburban boundaries and more than 50 m from human dwellings, with territories often including farmland [19]), time (hours since dawn), date (day of the year; representing the progression of the breeding season) and air temperature.

### 2.4. Nests, Eggs and Hatching Success

Once the experimental approach was completed, geographic coordinates (latitude and longitude) of the nest location were determined using a GPS, and the float stage for each egg (to estimate egg age) was recorded [15,30]. Each egg was individually numbered with a non-toxic permanent felt tip marker, so that they could be monitored individually during the study [15,31]. The relationship between egg flotation and the number of days to hatching was determined by a General Linear Model (GLM) [19]. To determine hatching success, nests were observed every 3–4 days to establish the fate of the eggs, with more frequent observations (every 2 days) around the predicted hatching date. Nests were judged to have ‘failed’ if they disappeared, were found destroyed or if ten days had elapsed since their predicted hatching date without the emergence of chicks. Nests were determined to have ‘succeeded’ only if at least one chick was seen completely out of the egg (eggs would occasionally begin to hatch, but the chick would not survive to emerge completely out of the egg). 

Ninety four nests were located between May 22 and September 15, 2012 (clutch size, 3.63 ± 1.07 (SD) eggs, 1–8 eggs; 75.5% of nests contained four eggs; see [32]). Of the 94 nests found, 69.9% produced at least one chick and were defined as successful. Of the 65 successful nests, 3.08 ± 0.91 chicks hatched per nest.

### 2.5. Data Analysis

For analysis, each nest was treated as an independent data point (studied nests were >50 m apart, but were generally hundreds of metres apart). In order to identify possible relationships between various behavioural measures, data were initially reduced by conducting principal component analysis (PCA) with varimax rotation (SPSS 20.0 [33,34,35]). Variables included in the PCA were: (1) the proportion of approach distance at which a response occurred (‘Response’), (2) the number of swoops during the approach above head height (‘Swoopabove’), (3) the number of swoops during approach below head height (‘Swoopbelow’), (4) the number of swoops during SOP above head height (‘Swoop2above’), (5) the number of swoops during SOP below head height (‘Swoop2below’), (6) the number of calls during the SOP (‘Calling’) and (7) the closest ground approach to the investigator (‘Proximity’ in metres). Principle components (hereafter ‘PC’) are numbered in decreasing order of their explanatory value (Table 1). ‘Swoops’ were defined as flight directed at the investigator by either bird.

**Table 1:** Component scores derived from a rotated principal component (PC) matrix of seven behavioural variables measured to characterise lapwing defence. Emboldened values indicate component scores of a magnitude ≥0.60, which were used to interpret the principal components.

Variables	Low swooping and calling (PC1)	High swooping (PC2)	Separation (PC3)
Percentage of Variance Explained (%)	34.0	22.9	16.3
Response	0.091	0.051	**0.834**
Swoopabove	0.124	**0.914**	−0.011
Swoopbelow	**0.826**	0.307	0.018
Swoop2above	0.092	**0.903**	0.053
Swoop2below	**0.860**	0.013	0.038
Calling	**0.667**	0.027	−0.035
Proximity	−0.231	−0.008	**0.773**

The first three principle components explained substantial amounts of variation in data (73.2%, Table 1). Thus, these components describe important aspects of the aggressive behaviour of nesting birds and represent the measures of different behaviours recorded. These principle components (PC1, PC2, PC3) were then used as response variables in General Linear Models (GLMs) to examine whether aggressive behaviour of nesting birds varied between stimuli (*i.e.*, approach types). PC1 and PC2 were log-transformed to meet the assumptions of normality; while PC3 already met this assumption and was not transformed. Univariate GLMs examined if the fixed factors of treatment (pedestrian/mower and eye stickers/no eye stickers) influenced defence (LogPC1, LogPC2 and PC3). GLMs were also used to determine if defence behaviour varied between habitats (suburban, n = 58, or rural, n = 19, or with season (day of the year)). Temperature, clutch age and laying date were included as covariates. Habitat was included as a random factor when analysing PC3, due to the significant relationship between habitat and aggression revealed by previous analysis (see Section 2.7).

Binary logistic regressions were used to determine if there was a relationship between hatching success (‘hatched’ (1) or ‘failed to hatch’ (0); response variable) and the continuous variables (defence variables (PC1, PC2 and PC3), progression of the breeding season (laying date), clutch age) and the fixed factor, habitat. Summary statistics are cited as the mean ± one standard deviation, unless otherwise indicated. 

### 2.6. Characterising Defence

From the seven variables included in the PCA, seven principal components were generated, three of which (PC1, PC2 and PC3) cumulatively accounted for 73.2% of variation in the data and were selected for further analyses. The rotated component matrix (Table 1) demonstrates the relationship between the principle components and the original variables, where original variables with component scores ≥0.60 are considered noteworthy. PC1 represents a gradient of calling and low swooping, with positive values reflecting more calling and low swooping (hereafter ‘low swooping and calling’). PC2 represents a gradient of high swooping, with positive values reflecting more high swooping (hereafter ‘high swooping’). PC3 represents behaviour characterised as a distraction, with positive values reflecting longer response distances and a greater minimum distance between the birds and the investigator during the SOP (hereafter ‘separation’). The three PCs identified describe the likely extent of human-lapwing conflict inherent in any response, where greatest conflict (*i.e.*, perceived ‘aggression’) is likely to be associated with high levels of low swooping, followed by intermediate conflict associated with high levels of high swooping, and minimal conflict is likely to be associated with higher levels of separation. Ultimately, the degree of conflict between lapwings and humans (and the human perceptions of lapwing aggressiveness) are human perceptions, which were not measured by this study.

### 2.7. Effect of Habitat and Egg Age on Defence

Habitat (suburban, 58 nests; rural, 19 nests), days to hatching, laying date (representing the progression of the breeding season) and temperature did not influence ‘low swooping and calling’ or ‘high swooping’. However, for ‘separation’, there was a significant effect of habitat, with higher component scores for rural birds compared with their suburban counterparts (rural birds responded earlier and remained further from the intruder; 0.88 ± 0.72 *vs*. −0.47 ± 0.82, Table 2). 

**Table 2:** Results from the General Linear Models investigating the influence of habitat, days to hatching, laying date (progression of the breeding season) and temperature on the parental defence variables (n = 77 nests). Significant values are emboldened. *Df* = degrees of freedom.

Response variable	Predictor variable	*Df*	F ratio	*P*
Low swooping and calling (Logged PC1)	Habitat	1, 72	0.005	0.942
R² = 0.050	Days to hatching	1, 72	0.384	0.528
	Laying date	1, 72	1.709	0.195
	Temperature (°C)	1, 72	0.343	0.560
High swooping (Logged PC2)	Habitat	1, 72	0.047	0.829
R² = 0.040	Days to hatching	1, 72	0.392	0.533
	Laying date	1, 72	2.087	0.153
	Temperature	1, 72	0.229	0.634
Separation (PC3)	Habitat	1, 72	31.809	< **0.001**
R² = 0.376	Days to hatching	1, 72	0.872	0.353
	Laying date	1, 72	1.111	0.295
	Temperature	1, 72	0.997	0.321

## 3. Results

### 3.1. Effect of Stimulus on Defence

**Figure 1:**
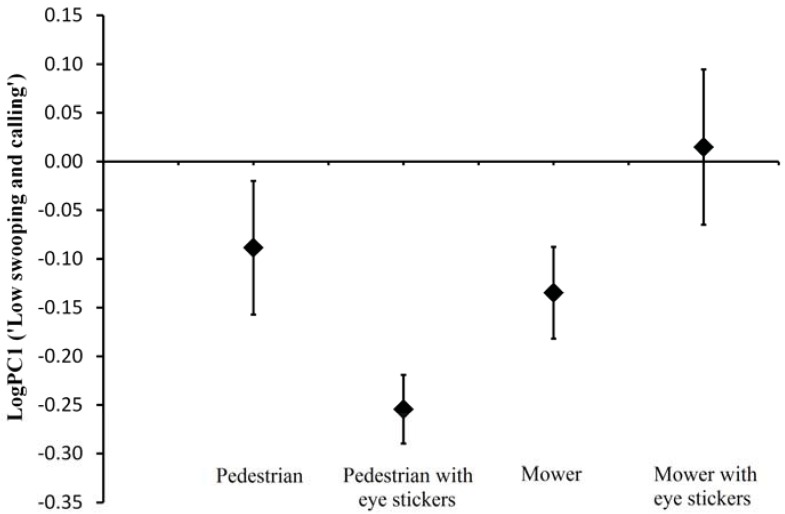
Means and one standard error of ‘low swooping and calling’ values against treatment type (the significant interaction between mower/pedestrian and eyes/no eyes; see Table 3). Means show the significant increase in mean ‘low swooping and calling’ values evoked by mowers with eye stickers when compared to mowers without eye stickers and the significant decrease in mean ‘low swooping and calling’ values when pedestrians without eye stickers are compared to pedestrians with eye stickers.

The presence of a lawn mower and the presence of eye stickers had no significant effect on ‘low swooping and calling’ or ‘high swooping’; however, there was a significant relationship between ‘separation’ and the presence of a lawn mower. Lawn mowers caused lapwings to flush earlier from the nest and remain further away from the stimulus. There was also a significant relationship between ‘low swooping and calling’ and the interaction between the presence of a lawn mower and the presence of eye stickers (Table 3; Figure 1). Thus, lapwings can discriminate between different human activities and adjust their defensive behaviour in regard to the perceived level of risk. Additionally, the use of eye stickers appears to be an effective method to mitigate the aggressive parental defence for pedestrians; however, eye stickers actually increase aggression directed towards those mowing lawns.

**Table 3:** Results of General Linear Models (GLMs) and General Linear Mixed Model (GLMM) (habitat included as a random effect for ‘separation’) investigating the influence of pedestrians *vs*. lawn mowers, eye stickers *vs*. no eye stickers, the interaction between pedestrians *vs*. lawn mowers and eye stickers *vs*. no eye stickers on parental defence variables (see Table 1). *Df* = degrees of freedom; significant values are emboldened.

Response variable	Predictor variable	*Df*	F ratio	*p*
Low swooping and calling (Logged PC1)	Mower/Pedestrian	1, 73	3.378	0.070
R² = 0.130	Eyes/No eyes	1, 73	0.018	0.895
	Mower/Pedestrian × Eyes/No eyes	1, 73	6.758	**0.011**
High swooping (Logged PC2)	Mower/Pedestrian	1, 73	0.165	0.686
R² = 0.012	Eyes/No eyes	1, 73	0.247	0.621
	Mower/Pedestrian × Eyes/No eyes	1, 73	0.639	0.427
Separation (PC3)	Mower/Pedestrian	1, 73	10.237	**0.002**
	Eyes/No eyes	1, 73	1.465	0.230
	Mower/Pedestrian × Eyes/No eyes	1, 73	1.362	0.247

### 3.2. Effect of Parental Defence on Hatching Success

‘Low swooping and calling’, ‘high swooping’, ‘separation’, habitat, days to hatching and laying date did not significantly influence hatching success (for all treatments pooled; Table 4); however, there was a tendency (*p* = 0.082) for longer ‘separation’ to be associated with higher hatching success.

**Table 4:** Results from six separate binary logistic regressions investigating the influence of ‘low swooping and calling’, ‘high swooping’, ‘separation’, habitat, days to hatching and laying date on hatching success (success; 0 = failed to hatch chicks, 1 = hatched at least one chick). *Df* = degrees of freedom; C = coefficient; SE = standard error; Z = test statistic (n = 85 nests).

Response variable	Predictor variable	*Df*	C	SE	Z	*P*
Success	Low swooping and calling	1, 71	−0.201	0.247	−0.81	0.415
	High swooping	1, 71	−0.103	0.181	−0.57	0.568
	Separation	1, 71	0.611	0.351	1.74	0.082
	Habitat	1, 71	1.164	0.795	1.46	0.143
	Days to hatching	1, 71	0.014	0.031	0.48	0.634
	Laying date	1, 71	−0.007	0.011	−0.67	0.505

## 4. Discussion

Like many birds [29,36,37], lapwings can distinguish between different human threats (*i.e.*, people with and without a lawn mower) and adjust their defence behaviour in regards to the perceived level of threat. We predicted that a person with a lawn mower would elicit more swooping and cause the defending birds to remain closer to the nest. Lawn mowers tended to elicit a more aggressive response (*i.e.*, more low swooping) from the birds than did pedestrians. Additionally, the lawn mower treatment was associated with lapwings remaining in closer proximity to the intruder. The widespread discrimination that birds exhibit between stimuli has been mostly demonstrated in their escape behaviour [18,36]; only rarely has it been demonstrated in aggressive behaviour directed towards humans [9,37]. Although well-studied, the aspects of stimuli used by birds to modify their responses to humans are still poorly understood; they apparently include speed, size, conspecific group size and angle of approach, all of which are presumably used to judge risk [29,38]. Lawn mowers may be perceived by lapwings as a greater threat to a nest, because clutches are frequently destroyed by lawn mowers on Phillip Island [39]. Thus, lapwings may have learned to attack them more vigorously. Attacks may also be mediated by the behaviour of the attacked (see [21,40]); pedestrians being swooped may alter their course or move away, while ground maintenance staff will generally continue mowing regardless of lapwing attacks. Lapwings may thus escalate their attacks given that they have not achieved their desired result (human retreat from the vicinity of a nest). This study did not examine human response to lapwing swooping, but the influence of human response to lapwing defence would be a fruitful area of future research.

This study appears to be the first to describe the discrimination of the attacking bird regarding the presence of an anti-swooping device (eye stickers) based on the context of the human who was wearing them. The presence of realistic looking eyes placed on the back of hats significantly decreased lapwing swooping of pedestrians, but interestingly increased the amount of swooping of a person who approached with a lawn mower. Several bird species are known to adjust their behaviour in relation to the direction of human gaze and even anticipate the direction in which a person is looking [21,22]. 

This study confirms the recommendations regarding the use of eye stickers by pedestrians to avoid lapwing swooping. However, it also suggests that the use of eye stickers on the back of the head of humans mowing lawns increased lapwing aggression and had the opposite effect to that intended. This emphasises the pitfalls associated with broad, untested management recommendations designed to manage complex and highly varied behavioural traits of wildlife, which may vary between places and species. Testing such management recommendations should perhaps be standard practice before advice is offered and include testing stimuli, which include all prominent human stakeholders (*i.e.*, stimuli). 

The significant relationship between ‘separation’ and habitat type suggests that nesting pairs in rural habitats flush earlier from the nest upon investigator approach and remain at a greater distance from the investigator during the SOP. This study suggests that pairs nesting in suburban environments may be more habituated to human presence or that local selection has influenced the responsiveness of local bird populations and is consistent with the finding of reduced flight initiation distances for many birds in areas of high human activity [18,41].

Contrary to our prediction, there was no significant relationship between parental defence and hatching success, but there was a tendency for larger ‘separation’ values to be associated with higher hatching success. Lapwings that do not advertise the presence of the nest by remaining close to it may avoid directing the attention of predators or people to their nest. Crypsis is employed by many species of ground-nesting bird and may have evolved, because predators have been selected to respond to obvious nests and the potential food source they contain [2,42,43]. However, crypsis may only work in certain circumstances. It is unclear why some birds approach closer, given that such behaviour is associated with lower hatching success. It is possible that birds that have previously suffered clutch loss exhibit closer approaches, either because their reproductive potential is more closely tied with defence (e.g., predators are abundant locally or they are more likely to be defending replacement clutches) or they have learned to become more aggressive, because they have defended clutches previously without being harmed. Adults may also be trading off their survival with that of their eggs [2,3]; so, those birds approaching more closely may have different reproductive potentials to those that remain further away. 

## 5. Conclusion

Lapwings can discriminate between different, common anthropogenic stimuli, directing more aggression at those mowing lawns than those walking by. This study found eye stickers decreased (pedestrians) and increased (people pushing lawn mowers) swooping behaviour. The results suggest that lapwings make complex decisions regarding risk to their nests and adjust their defensive behaviour accordingly. Existing recommendations by management authorities regarding the use of eye stickers by pedestrians to deter lapwing swooping appear efficacious. However, eye stickers were counterproductive for ground maintenance workers and different management solutions are required to reduce swooping in these circumstances. 
